# Predictors of atrial fibrillation detection in embolic stroke of undetermined source patients with implantable loop recorder

**DOI:** 10.3389/fcvm.2024.1369914

**Published:** 2024-03-04

**Authors:** Lucio D’Anna, Roberta La Cava, Ashni Khetarpal, Abeer Karjikar, Ahmad Almohtadi, Michele Romoli, Matteo Foschi, Raffaele Ornello, Federico De Santis, Simona Sacco, Samir Abu-Rumeileh, Simone Lorenzut, Daisy Pavoni, Mariarosaria Valente, Giovanni Merlino, Soraia Almeida, Asha Barnard, Jianqun Guan, Soma Banerjee, Phang Boon Lim

**Affiliations:** ^1^ Department of Stroke and Neuroscience, Charing Cross Hospital, Imperial College London NHS Healthcare Trust, London, United Kingdom; ^2^ Department of Brain Sciences, Imperial College London, London, United Kingdom; ^3^ Neurology and Stroke Unit, Department of Neuroscience, Bufalini Hospital, AUSL Romagna, Cesena, Italy; ^4^ Department of Biotechnological and Applied Clinical Sciences, University of L’Aquila, L’Aquila, Italy; ^5^ Department of Neurology, Martin-Luther-University Halle-Wittenberg, Halle (Saale), Germany; ^6^ Stroke Unit, Udine University Hospital, Udine, Italy; ^7^ Cardiothoracic Department, Udine University Hospital, Udine, Italy; ^8^ Clinical Neurology, Udine University Hospital and DAME, University of Udine, Udine, Italy; ^9^ Stroke Unit and Clinical Neurology, Udine University Hospital, Udine, Italy; ^10^ Department of Cardiology, Hammersmith Hospital, Imperial College London NHS Healthcare Trust, London, United Kingdom

**Keywords:** embolic stroke of undetermined source, loop recorder, ischemic stroke, atrial flutter, atrial fibrillation

## Abstract

**Background:**

Covert atrial fibrillation (AF) is a predominant aetiology of embolic stroke of undetermined source (ESUS). Evidence suggested that AF is more frequently detected by implantable loop recorder (ILR) than by conventional monitoring. However, the predictive factors associated with occult AF detected using ILRs are not well established yet. In this study we aim to investigate the predictors of AF detection in patients with ESUS undergoing an ILR.

**Methods:**

This observational multi-centre study included consecutive ESUS patients who underwent ILR implantation. The infarcts were divided in deep, cortical infarcts or both. The infarction sites were categorized as anterior and middle cerebral artery, posterior cerebral artery with and without brainstem/cerebellum involvement. Multivariable logistic regression analysis was performed to investigate variables associated with AF detection.

**Results:**

Overall, 3,000 patients were initially identified. However, in total, 127 patients who consecutively underwent ILR implantation were included in our analysis. AF was detected in 33 (26%) out of 127 patients. The median follow-up was 411 days. There were no significant differences in clinical characteristics and comorbidities between patients with and without AF detected. AF was detected more often after posterior cerebral artery infarct with brainstem/cerebellum involvement (*p* < 0.001) whereas less often after infarction in the anterior and middle cerebral artery (*p* = 0.021). Multivariable regression analysis demonstrated that posterior cerebral artery infarct with brainstem/cerebellum involvement was an independent predictor of AF detection.

**Conclusion:**

Our study showed that posterior circulation infarcts with brainstem/cerebellum involvement are associated with AF detection in ESUS patients undergoing ILR. Larger prospective studies are needed to validate our findings.

## Introduction

Atrial fibrillation (AF) and atrial flutter can be newly detected in approximately one-fourth of patients with ischemic stroke and transient ischemic attack without previously recognised AF (1). Oral anticoagulation is recommended by American and European guidelines to reduce the risk of stroke and systemic embolism in stroke patients with AF (2, 3). However, previous randomised controlled trials (RCTs) on embolic stroke with undetermined source (ESUS) suggested that empiric anticoagulation following the event is not proven to be a good strategy (4, 5). Therefore, there is the need to perform additional prolonged cardiac monitoring to detect AF that may help guide the choice of optimal antithrombotic therapy. Several RCTs have proven that implantable loop recorder (ILR) was superior to conventional monitoring for detecting AF after cryptogenic stroke (6–8). Moreover, further meta-analysis of RCTs was conducted to quantify the incremental change in AF detection and the subsequent risk of stroke associated with ILR use vs. usual care in post-stroke settings (9). However, despite ILR was superior to usual care in AF detection, there was a non-differential risk of stroke between the ILR and usual care arms. This might indicate that some of the ESUS patients do not benefit from ILR implantation and since use of an ILR is relatively expensive and invasive, further studies are warranted to understand how patient selection can be improved to increase the diagnostic yield of ILR. Our prospective multicentre study aimed to investigate the neuroimaging patterns and clinical characteristics associated with AF detection in patients with ESUS undergoing a ILR in the setting of a multicentre study.

## Methods

**Figure 1:**
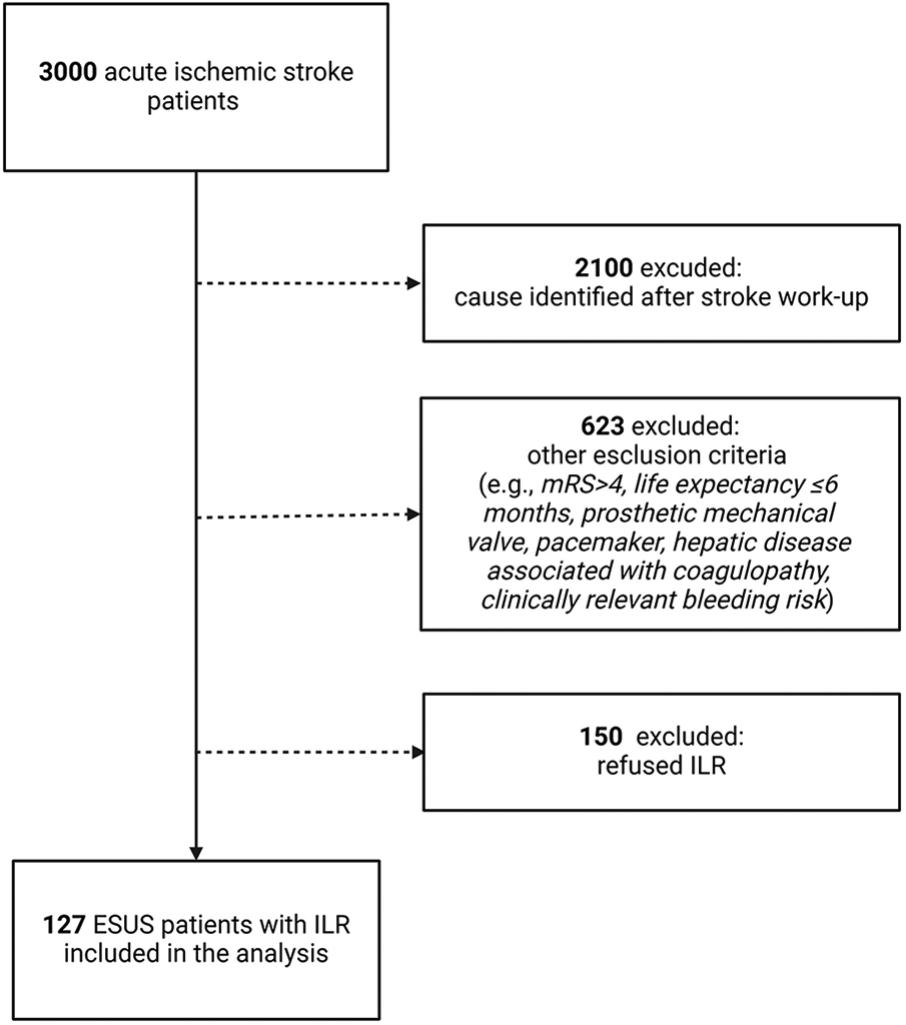
Study flow chart.

This was a prospective, multicentre, observational, investigator-initiated study, that included consecutive patients who underwent ILR (Reveal LINQ, Medtronic Inc, Minneapolis, MN) implantation after a diagnosis of ESUS and without history of AF or atrial flutter between 1st January 2018 and 31st December 2021. Patients were recruited from the ILR registry of the following hospitals: Stroke Department, Charing Cross Hospital, Imperial College Healthcare NHS Trust, London; Stroke Unit, Department of Neuroscience, Bufalini Hospital, AUSL Romagna, Cesena, Italy; Neurology Department, Udine University Hospital, Udine, Italy; Stroke Unit, Avezzano Hospital, Avezzano, Italy. The study was conducted in accordance with the recommendations for physicians involved in research on human subjects adopted by the 18th World Medical Assembly, Helsinki 1964 and later revisions. This study has obtained approval from the UK Health Regulator Authority (Health Regulator Authority Reference No.: 275260). ESUS was defined according to the criteria proposed by the Cryptogenic Stroke/ESUS International Working Group, as follows: (1) stroke detected by magnetic resonance imaging that is not lacunar, (2) absence of extracranial or intracranial stenosis causing ≥50% luminal stenosis in arteries supplying the area of ischemia detected with a magnetic resonance angiography (MRA) or computed tomography angiogram (CTA), (3) no major-risk cardioembolic source, and (4) no other specific cause of stroke identified (e.g., arteritis, dissection, vasospasm, drug misuse) (10). For this analysis, we excluded patients with a modified Rankin Scale (mRS) post-stroke >4, life expectancy less than 6 months, prosthetic mechanical valve, pacemaker, hepatic disease associated with coagulopathy (prothrombin time prolonged beyond the normal range) and clinically relevant bleeding risk including cirrhotic patients with Child Pugh B and C and estimated glomerular filtration rate (eGFR) < 15 ml/min/1.73 m^2^ (Study flow chart, Figure 1). Twelve-lead electrocardiography, transthoracic or transoesophageal echocardiography, and cardiac monitoring for at least 24 h were performed before determining the indication for ILR implantation. Data of consecutive patients who underwent ILR implantation were collected prospectively and encompassed patient characteristics, including age, vascular risk factors, relevant medical history. NIHSS was performed in all patients on admission. The mRS was used to assess the patient's functional status post-stroke and before determining the indication for ILR implantation and was evaluated through an in-person consultation. Topographically, the infarcts were divided in deep infarcts as entirely subcortical, cortical infarcts when involving the cortex or both. The infarction sites were categorized as anterior and middle cerebral artery (internal carotid artery, ophthalmic artery, anterior cerebral artery, middle cerebral artery and internal carotid artery sub-territories) and posterior cerebral artery with and without brainstem/cerebellum involvement (vertebral and basilar artery territories). After written informed consent, the patients underwent ILR implantation under local anesthesia. The patients were routinely followed up in the outpatient clinic, and the detection of AF was evaluated. AF was defined as an episode of irregular hearth rhythm, without detectable *P* waves, lasting more than 30 s. Time variables were collected prospectively and included day of stroke onset, ILR implantation and first AF detection.

Statistical analyses were performed with R software, version 4.2.2. Descriptive categorical data were reported as numbers and proportions; descriptive continuous data were reported as means and standard deviations (SDs) for normally distributed variables, including age and blood pressure values, or medians and interquartile ranges (IQRs) for non-normally distributed variables, including stroke scale scores. We compared the demographic, clinical and neuroimaging characteristics of the two groups (no AF vs. new-onset AF) by chi-square test (for categorical variables), one-way ANOVA (for normally distributed continuous variables, followed by Tukey's *post hoc* test), or Kruskal-Wallis test (for non-normally distributed continuous variables followed by the Dunn-Bonferroni *post hoc* test). *P* values were considered statistically significant at <0.05. We performed a univariable logistic regression analysis with calculation of odds ratios (ORs) and 95% confidence intervals (Cis) to investigate variables associated with AF detection. Variables with an association with AF detection (*P* ≤ 0.05) were considered for multivariable logistic regression analysis with statistical significance set at a *P* < 0.05. This study was approved by the local institutional review boards. All authors had full access to the data and have read and agreed to the article as written.

## Results

Overall, 3,000 patients were initially identified (Study flow chart, Figure 1). However, in total, 127 patients who consecutively underwent ILR implantation were included in our analysis. AF was detected in 33 (26%) out of 127 patients. Demographic and clinical features of the patients are reported in Table 1. There were no significant differences in clinical characteristics, comorbidities, and admission therapy between patients with and without AF. The duration of the cardiac monitoring pre-ILR implantation did not significantly differ between patients with and without AF, respectively 5 (IQR, 2–7) days and 3 (IQR, 2–7) days, (*p* = 0.471). The median duration of ILR monitoring for our patient sample was 411 (IQR, 274–624) days (Supplementary Table S1) and did not differ significantly between patients with and without AF detection (*p* = 0.567). AF was detected from the stroke onset with a median interval time of 353 (IQR, 182–740) days; and from the ILR implantation with a median interval time of 71 (IQR, 58–250) days. The neuroimaging characteristics according to the AF detection are shown in Table 2. The two groups did not differ in terms of infarct number and infarct location, (respectively, *p* = 0.769 and *p* = 0.124). AF was detected less frequently in patients who had infarction in the anterior and middle cerebral artery (*p* = 0.021) whereas more often in patients with posterior cerebral artery with brainstem/cerebellum involvement (*p* < 0.001).

**Table 1:** Baseline characteristics.

	Patients (*n* = 127)	Patients AF detection (*n* = 33)	Patients with no AF detection (*n* = 94)	*p*-value
Demographics				
Age, years [mean ± standard deviation]	61.0 ± 11.5	61.5 ± 12.4	60.8 ± 11.3	0.514
Female sex [*n*, (%)]	78 (61.4)	21 (63.6)	57 (60.6)	0.923
Cardiovascular risk factors				
Hypertension [*n*, (%)]	53 (42)	17 (51.5)	36 (61.7)	0.413
Diabetes mellitus [*n*, (%)]	18 (14.2)	5 (15.2)	13 (13.8)	1
Hypercholesterolemia [*n*, (%)]	43 (33.9)	11 (33.3)	32 (34.0)	0.832
Current smoking, [*n*, (%)]	49 (38.6)	12 (36.3)	37 (39.4)	0.923
Coronary artery disease [*n*, (%)]	20 (11.6)	5 (15.2)	15 (15.9)	0.985
Congestive heart failure [*n*, (%)]	10 (7.8)	3 (9.1)	7 (7.4)	0.532
Type of cerebrovascular event:				0.569
TIA *n*, (%)]	21 (16.5)	7 (21.2)	14 (14.9)	
Ischemic stroke [*n*, (%)]	106 (83.5)	26 (78.9)	80 (85.1)	
Malignancy [*n*, (%)]	11 (8.7)	5 (15.2)	6 (6.4)	0.237
Total CHA2DS2 VASC score [median (IQR)]	4 (3–5)	4 (2.25–5)	4 (3,4)	0.515
Previous Stroke/TIA [*n*, (%)]	41 (32.3)	10 (30.3)	31 (32.3)	0.947
Admission therapy				
Antiplatelet therapy on admission [*n*, (%)]	43 (33.9)	11 (33.3)	32 (34.0)	1
NIHSS on admission [median (IQR)]	2 (0.25–4)	3 (1–5)	2 (0–4)	0.271

**Table 2:** Neuroimaging characteristics.

	Patients AF detection	Patients no AF detection	*p*-value
	(*n* = 33)	(*n* = 94)	
Infarct number, [*n*, (%)]			0.769
Single,	16 (48.5)	50 (53.2)	
Multiple	17 (51.5)	44 (46.8)	
Infarct location, [*n*, (%)]			0.124
Deep	15 (45.5)	12 (12.8)	
Cortical	15 (45.5)	51 (54.3)	
Both	3 (9)	6 (32.9)	
Infarct Site, [*n*, (%)]			
Anterior and Middle cerebral artery	21 (63.6)	78 (82.9)	0.021
Posterior cerebral artery	7 (21.2)	19 (20.2)	0.906
Posterior cerebral artery and brainstem/cerebellum	19 (57.6)	37 (39.4)	<0.001

**Figure 2:**
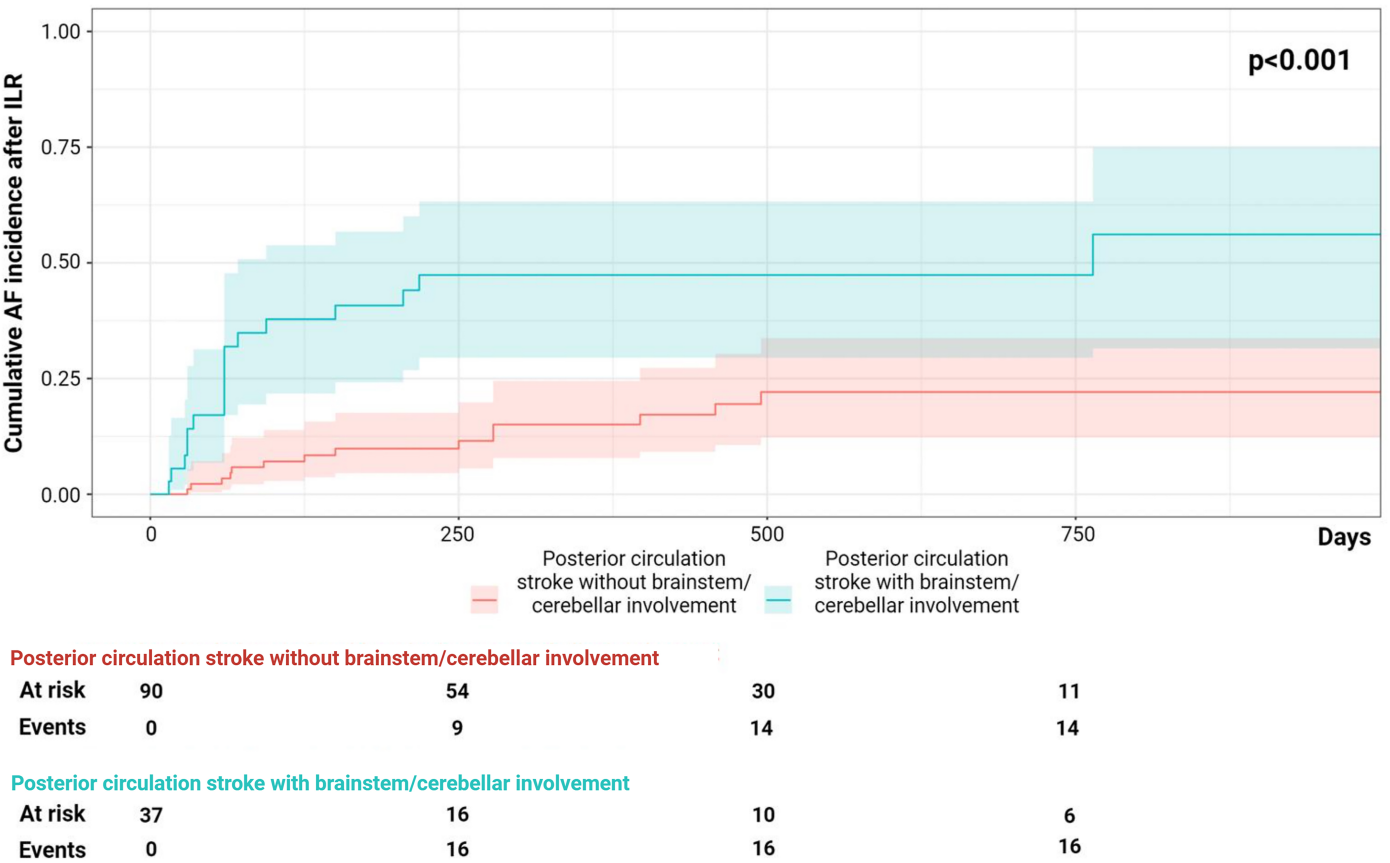
The cumulative incidence analysis on atrial fibrillation (AF) detection during follow-up, posterior circulation infarct with and without brainstem/cerebellum involvement.

Patients with detected AF compared to those without AF detection did not differ in terms of characteristics at the echocardiogram (Table 3). Table 4 shows the results of the univariate and multivariable logistic regression analyses. Multivariable regression analysis showed that posterior circulation infarct with brainstem/cerebellum involvement was an independent predictor of AF detection in ESUS patients (OR 1.22, CI 1.57–7.38, *p* = 0.02). The cumulative incidence analysis demonstrated that the patients with ESUS in the posterior circulation with brainstem/cerebellum involvement showed a higher AF detection rate compared with the patients with ESUS not in this location (log-rank, *p* = <0.001; Figure 2).

**Table 3:** Characteristics at the echocardiogram.

	Patients AF detection (*n* = 33)	Patients no AF detection (*n* = 94)	*p*-value
Left atrium dilatation, [*n*, (%)]	12 (36.4)	21 (22.3)	0.188
Mitral valve pathology, [*n*, (%)]	7 (21.2)	21 (22.3)	0.974
Aortic valve pathology, [*n*, (%)]	6 (18.2)	17 (18.1)	1
Presence of supraventricular tachycardia, [*n*, (%)]	8 (24.2)	29 (30.9)	0.627
Presence of atrial ectopics, [*n*, (%)]	19 (57.6)	57 (60.6)	0.798

**Table 4:** Result of univariable and multivariable analysis for detection of atrial fibrillation in ESUS patients.

	Univariable analysis		Multivariable analysis	
	OR (95% CI)	*p*-value	OR (95% CI)	*p*-value
Age	1.00 (0.98–1.04)	0.519	-	-
Gender sex			-	-
Male	1 reference	0.358		
Female	0.70 (0.33–1.50)			
Hypertension	0.71 (0.36–1.41)	0.334	-	-
Diabetes mellitus	1.06 (0.41–2.76)	0.898	-	-
Hypercholesterolemia	1.08 (0.52–2.26)	0.821	-	-
Current smoking	0.86 (0.42–1.76)	0.689	-	-
Coronary artery disease	0.97 (0.38–2.53)	0.961	-	-
Congestive heart failure	0.35 (0.40–1.54)	0.996	-	-
Type of cerebrovascular event:			-	-
TIA	1 reference	0.339		
Ischemic stroke	0.66 (0.29–1.53)			
mRS pre-stroke per one point	0.93 (0.58–1.48)	0.767	-	
Malignancy	1.96 (0.75–5.12)	0.169	-	-
Total CHA2DS2 VASC score per one point	1.02 (0.76–1.37)	0.877	-	-
Previous Stroke/TIA	0.87 (0.41–1.85)	0.727	-	-
Antiplatelet therapy on admission	1.22 (0.58–2.54)	0.599	-	-
NIHSS on admission per one point	1.05 (0.99–1.11)	0.098	-	-
Duration of ILR monitoring per day	0.99 (0.99–1.00)	0.136	-	-
Infarct number			-	-
Single	1.38 (0.31–6.12)	0.669		
Multiple	1.61 (0.37–7.00)	0.525		
Infarct location			-	-
Deep	1 reference			
Cortical	3.28 (1.51–7.10)	0.997		
Both	2.24 (0.90–5.57)	0.997		
Anterior and Middle cerebral artery	0.36 (0.17–0.74)	0.009	0.66 (0.29–1.49)	0.319
Posterior cerebral artery	1.01 (0.44–2.33)	0.978	-	-
Posterior cerebral artery and brainstem/cerebellum	4.01 (2.01–8.01)	<0.001	1.22 (1.57–7.38)	0.002
Left atrium dilatation	1.95 (0.95–3.97)	0.065	-	-
Mitral valve pathology	0.91 (0.39–2.12)	0.832	-	-
Aortic valve pathology	0.85 (0.34–2.08)	0.721	-	-
Presence of supraventricular tachycardia	0.91 (0.40–2.06)	0.826	-	-
Presence of atrial ectopics	1.07 (0.53–2.19)	0.844	-	-
Duration of the Holter pre ILR per day	1.02 (0.94–1.10)	0.595	-	-

## Discussion

The main original finding of our study is that the presence of infarcts in the posterior circulation with brainstem/cerebellum involvement was associated with AF detection in our ESUS cohort. To our knowledge, this is the first analysis that documented a strong relationship between posterior circulation infarcts with brainstem/cerebellum involvement in ESUS patients and detection of AF after ILR implantation. Previous studies have investigated predictive factors associated with AF detection using ILR in patients with cryptogenic or ESUS stroke. Older age (11), diabetes (12), left atrial enlargement (13), higher CHA2D2-VASC score (14), higher body mass index (15), N-terminal prohormone of brain natriuretic peptide (13, 16, 17), troponin T at baseline (15) were found to be independently associated with AF detection. In contrast to previous studies, our main focus was to identify the neuroimaging patterns associated with the diagnostic yield of AF in patients with ESUS who received ILR. Yushan et al. found that a neuroimaging profile of bilateral infarcts was associated with AF detection using insertable cardiac monitor in ESUS patients (18) while Kim et al. demonstrated a higher AF detection rate associated with whole-territory infarction on brain imaging (19). Makimoto et al. (20) previously reported that posterior cerebral artery stroke but not in the territory of the vertebral artery may be more frequently related to AF than other stroke localizations in ESUS. In our study a higher percentage of ESUS patients experienced a posterior circulation stroke compared to the analysis of Makimoto et al. It is noteworthy to mention that, in contrast to Makimoto et al., all our ESUS patients underwent magnetic resonance imaging (MRI) in combination with a computer tomography (CT) scan. MRI is considered more sensitive than CT to detect posterior circulation infarcts and this might explain the differences between the two studies.

Based on our findings, we could not clarify the mechanism of higher AF detection in patients with posterior circulation infarcts with brainstem/cerebellum involvement. Nevertheless, it is noteworthy to mention the hypothetical pathophysiological model according to which AF detected after an acute ischemic stroke may be short-lasting and perhaps a nonrecurrent autonomic and inflammatory epiphenomena of stroke (21). The autonomic regulation of cardiac rhythm constitutes an integrated relay system represented by the insula, hypothalamus, limbic system, and brainstem nuclei (22). The onset of AF may be associated with an imbalance between sympathetic and parasympathetic activities, as the consequence of a brain infarct in one of these strategic points of the relay system (23). However, to what extent poststroke AF is the cause or a consequence remains uncertain to date.

Our study was able also to confirm the utility of ILRs in clinical practice for stroke investigation. In the CRYSTAL-AF trial (8) the median time to AF detection was 84 days. The results of our study were consistent with the findings of the CRYSTAL-AF trial, with our median time of 71 days to detect AF. Conversely, the rate of detection of AF in our study was more in line with other observational studies, showing rates upwards of 25% using ILRs (14). This difference could possibly be due to differences in the selection and assessment of patients between the studies. Our analysis suggests the need to identify which ESUS patients can benefit the most from ILR insertion, and future studies focusing on anticoagulation therapy after ESUS should encompass patients with a high probability of covert AF, rather than all ESUS patients. Indeed, previous trials might have recruited a heterogenous group of patients who might not have a cardioembolic infarction. Thus, considering the results of our study, future clinical trials should focus on analysing patient clinical features and neuroimaging patterns.

### Limitations

The strengths of the present study include its multicentre design including data from prospective stroke registries and the relative long follow-up duration after ILR implantation. Nevertheless, this study has a few possible limitations which may impact study results. Our data were collected prospectively but this was a retrospective analysis, therefore it is important to note that associations found do not imply causal relationships. While there was no statistically significant difference in terms of average follow-up time between the two groups, it may not have been long enough to catch an arrhythmic episode causing stroke. Moreover, given the nature of the study we cannot exclude a selection bias. However, we used a consecutive enrolment and applied strict inclusion/exclusion criteria. Therefore, we believe the validity of our data in the present study.

### Future directions

Our data highlight the importance of ESUS location to identify the best candidate for ILR insertion. We believe that there is a paramount need of future larger prospective studies are needed to validate our findings in larger multicentre cohorts.

## Conclusions

Our study showed that a neuroimaging profile of posterior circulation infarct with brainstem/cerebellum involvement was associated with AF detection using ILR in ESUS patients.

## Data Availability

The raw data supporting the conclusions of this article will be made available by the authors, upon reasonable request.
