# Supplementary material for: Predictors of atrial fibrillation detection in embolic stroke of undetermined source patients with implantable loop recorder
Source: Front Cardiovasc Med. 2024 Mar 4;11:1369914. doi: 10.3389/fcvm.2024.1369914 (PMC10944994; doi:10.3389/fcvm.2024.1369914)
Supplement: Supplementary file 1 [file Table1.docx]

**Supplemental Table 1. Time intervals.**

|  | **Patients with AF detected (n = 33)** | **Patients with no AF detected**  **(n = 94)** | **p-value** |
| --- | --- | --- | --- |
| **Duration of ILR monitoring, (days)**  **[median (IQR)**] | 411 (274-624) | 378 (178.5-618.5) | 0.567 |
| **Interval stroke-AF detection, (days)**  **[median (IQR)**] | 353 (182-740) | **-** | **-** |
| **Interval ILR implant-AF detection, (days)**  **[median (IQR)**] | 71 (58-250) | **-** | **-** |
