# Supplementary material for: Predictors of atrial fibrillation detection in embolic stroke of undetermined source patients with implantable loop recorder
Source: Front Cardiovasc Med. 2024 Mar 4;11:1369914. doi: 10.3389/fcvm.2024.1369914 (PMC10944994; doi:10.3389/fcvm.2024.1369914)
Supplement: Supplementary file 2 [file Table2.docx]

**Predictors of atrial fibrillation detection in patients with Embolic Stroke of Undetermined Source**

All analyses were performed with R software, version 4.2.2.

**Scripts**

**#Descriptive statistics overall population**

**Packages:** library(stats), library(dplyr)

options(scipen=999)

paste(mean(stroke_ilr$age), "(", sd(stroke_ilr$age),")")

paste(median(stroke_ilr$admission.nihss), "(", quantile(stroke_ilr$admission.nihss, 0.25), "-", quantile(stroke_ilr$admission.nihss, 0.75), ")")

paste(median(stroke_ilr$days.stroke.ilr), "(", quantile(stroke_ilr$days.stroke.ilr, 0.25), "-", quantile(stroke_ilr$days.stroke.ilr, 0.75), ")")

paste(median(stroke_ilr$days.stroke.af), "(", quantile(stroke_ilr$days.stroke.af, 0.25), "-", quantile(stroke_ilr$days.stroke.af, 0.75), ")")

paste(median(stroke_ilr$days.total), "(", quantile(stroke_ilr$days.total, 0.25), "-", quantile(stroke_ilr$days.total, 0.75), ")")

paste(median(stroke_ilr$days.ilr.af), "(", quantile(stroke_ilr$days.ilr.af, 0.25), "-", quantile(stroke_ilr$days.ilr.af, 0.75), ")")

paste(median(stroke_ilr$ilr.duration), "(", quantile(stroke_ilr$ilr.duration, 0.25), "-", quantile(stroke_ilr$ilr.duration, 0.75), ")")

paste(median(stroke_ilr$time.after.stroke), "(", quantile(stroke_ilr$time.after.stroke, 0.25), "-", quantile(stroke_ilr$time.after.stroke, 0.75), ")")

paste(median(stroke_ilr$time.after.ilr), "(", quantile(stroke_ilr$time.after.ilr, 0.25), "-", quantile(stroke_ilr$time.after.ilr, 0.75), ")")

paste(median(stroke_ilr$chad), "(", quantile(stroke_ilr$chad, 0.25), "-", quantile(stroke_ilr$chad, 0.75), ")")

paste(median(stroke_ilr$bmi), "(", quantile(stroke_ilr$bmi, 0.25), "-", quantile(stroke_ilr$bmi, 0.75), ")")

paste(median(stroke_ilr$duration.holter), "(", quantile(stroke_ilr$duration.holter, 0.25), "-", quantile(stroke_ilr$duration.holter, 0.75), ")")

paste(median(stroke_ilr$mrs.discharge), "(", quantile(stroke_ilr$mrs.discharge, 0.25), "-", quantile(stroke_ilr$mrs.discharge, 0.75), ")")

paste(median(READAPT$Age), "(", quantile(READAPT$Age, 0.25), "-", quantile(READAPT$Age, 0.75), ")")

paste(median(stroke_ilr$mrs.180), "(", quantile(stroke_ilr$mrs.180, 0.25), "-", quantile(stroke_ilr$mrs.180, 0.75), ")")

**#AF yes vs. AF no**: **categoric variables**

table(filter(stroke_ilr,af==0)$sex)

table(filter(stroke_ilr,af==1)$sex)

table(stroke_ilr$sex)

table(filter(stroke_ilr,af==0)$hypertension)

table(filter(stroke_ilr,af==1)$hypertension)

table(stroke_ilr$center)

table(filter(stroke_ilr,af==0)$center)

table(filter(stroke_ilr,af==1)$center)

table(stroke_ilr$cholest)

table(filter(stroke_ilr,af==0)$ethnic)

table(filter(stroke_ilr,af==1)$ethnic)

table(stroke_ilr$ethnic)

table(filter(stroke_ilr,af==0)$mrs.prestroke)

table(filter(stroke_ilr,af==1)$mrs.prestroke)

table(stroke_ilr$mRS.prestroke)

table(filter(stroke_ilr,af==0)$strokevsTIA)

table(filter(stroke_ilr,af==1)$strokevTIA)

table(stroke_ilr$strokevsTIA)

table(filter(stroke_ilr,af==0)$prev.antiplatelet)

table(filter(stroke_ilr,af==1)$prev.antiplatelet)

table(stroke_ilr$prev.antiplatelet)

table(filter(stroke_ilr,af==0)$prev.stroke.tia)

table(filter(stroke_ilr,af==1)$prev.stroke.tia)

table(stroke_ilr$prev.stroke.tia)

table(filter(stroke_ilr,af==0)$icd)

table(filter(stroke_ilr,af==1)$icd)

table(stroke_ilr$icd)

table(filter(stroke_ilr,af==0)$diabetes)

table(filter(stroke_ilr,af==1)$diabetes)

table(stroke_ilr$diabetes)

table(filter(stroke_ilr,af==0)$chf)

table(filter(stroke_ilr,af==1)$chf)

table(stroke_ilr$chf)

table(filter(stroke_ilr,af==0)$chf)

table(filter(stroke_ilr,af==1)$chf)

table(stroke_ilr$chf)

table(filter(stroke_ilr,af==0)$cancer)

table(filter(stroke_ilr,af==1)$cancer)

table(stroke_ilr$cancer)

table(filter(stroke_ilr,af==0)$smoking)

table(filter(stroke_ilr,af==1)$smoking)

table(stroke_ilr$smoking)

table(filter(stroke_ilr,af==0)$cholest)

table(filter(stroke_ilr,af==1)$cholest)

table(stroke_ilr$cholest)

table(filter(stroke_ilr,af==0)$imaging.type)

table(filter(stroke_ilr,af==1)$imaging.type)

table(stroke_ilr$imaging.type)

table(filter(stroke_ilr,af==0)$imaging.vessel)

table(filter(stroke_ilr,af==1)$imaging.vessel)

table(stroke_ilr$imaging.vessel)

table(filter(stroke_ilr,af==0)$location)

table(filter(stroke_ilr,af==1)$location)

table(stroke_ilr$location)

table(filter(stroke_ilr,af==0)$infarct)

table(filter(stroke_ilr,af==1)$infarct)

table(stroke_ilr$infarct)

table(filter(stroke_ilr,af==0)$infarct.number)

table(filter(stroke_ilr,af==1)$infarct.number)

table(stroke_ilr$infarct.number)

table(filter(stroke_ilr,af==0)$ant.post)

table(filter(stroke_ilr,af==1)$ant.post)

table(stroke_ilr$ant.post)

table(filter(stroke_ilr,af==0)$subtentorial)

table(filter(stroke_ilr,af==1)$subtentorial)

table(stroke_ilr$subtentorial)

table(filter(stroke_ilr,af==0)$left.atrium.abnormal)

table(filter(stroke_ilr,af==1)$left.atrium.abnormal)

table(stroke_ilr$left.atrium.abnormal)

table(filter(stroke_ilr,af==0)$mitral.abnormal)

table(filter(stroke_ilr,af==1)$mitral.abnormal)

table(stroke_ilr$mitral.abnormal)

table(filter(stroke_ilr,af==0)$aortic.abnormal)

table(filter(stroke_ilr,af==1)$aortic.abnormal)

table(stroke_ilr$aortic.abnormal)

table(filter(stroke_ilr,af==0)$svt)

table(filter(stroke_ilr,af==1)$svt)

table(stroke_ilr$svt)

table(filter(stroke_ilr,af==0)$ectopics)

table(filter(stroke_ilr,af==1)$ectopics)

table(stroke_ilr$ectopics)

table(filter(stroke_ilr,af==0)$mrs.discharge.bad)

table(filter(stroke_ilr,af==1)$mrs.discharge.bad)

table(stroke_ilr$mrs.discharge.bad)

table(filter(stroke_ilr,af==0)$mrs.90.bad)

table(filter(stroke_ilr,af==1)$mrs.90.bad)

table(stroke_ilr$mrs.90.bad)

table(filter(stroke_ilr,af==0)$mrs.180.bad)

table(filter(stroke_ilr,af==1)$mrs.180.bad)

table(stroke_ilr$mrs.180.bad)

table(filter(stroke_ilr,af==0)$stroke.recurrent.90)

table(filter(stroke_ilr,af==1)$stroke.recurrent.90)

table(stroke_ilr$stroke.recurrent.90)

table(filter(stroke_ilr,af==0)$stroke.recurrent.180)

table(filter(stroke_ilr,af==1)$stroke.recurrent.180)

table(stroke_ilr$stroke.recurrent.180)

table(filter(stroke_ilr,af==0)$cv.90)

table(filter(stroke_ilr,af==1)$cv.90)

table(stroke_ilr$cv.90)

table(filter(stroke_ilr,af==0)$cv.180)

table(filter(stroke_ilr,af==1)$cv.180)

table(stroke_ilr$cv.180)

table(filter(stroke_ilr,af==0)$composite.90)

table(filter(stroke_ilr,af==1)$composite.90)

table(stroke_ilr$composite.90)

table(filter(stroke_ilr,af==0)$composite.180)

table(filter(stroke_ilr,af==1)$composite.180)

table(stroke_ilr$composite.180)

**#AF yes vs. AF no: continuous variables**

paste(median(filter(stroke_ilr,af==1)$age),"(",quantile(filter(stroke_ilr,af==1)$age,0.25),"-",quantile(filter(stroke_ilr,af==1)$age,0.75),")")

paste(mean(filter(stroke_ilr,af==1)$age), "(", sd(filter(stroke_ilr,af==1)$age),")")

paste(median(filter(stroke_ilr,af==1)$admission.nihss),"(",quantile(filter(stroke_ilr,af==1)$admission.nihss,0.25),"-",quantile(filter(stroke_ilr,af==1)$admission.nihss,0.75),")")

paste(median(filter(stroke_ilr,af==1)$days.stroke.ilr),"(",quantile(filter(stroke_ilr,af==1)$days.stroke.ilr,0.25),"-",quantile(filter(stroke_ilr,af==1)$days.stroke.ilr,0.75),")")

paste(median(filter(stroke_ilr,af==1)$days.stroke.af),"(",quantile(filter(stroke_ilr,af==1)$days.stroke.af,0.25),"-",quantile(filter(stroke_ilr,af==1)$days.stroke.af,0.75),")")

paste(median(filter(stroke_ilr,af==1)$days.total),"(",quantile(filter(stroke_ilr,af==1)$days.total,0.25),"-",quantile(filter(stroke_ilr,af==1)$days.total,0.75),")")

paste(median(filter(stroke_ilr,af==1)$ilr.duration),"(",quantile(filter(stroke_ilr,af==1)$ilr.duration,0.25),"-",quantile(filter(stroke_ilr,af==1)$ilr.duration,0.75),")")

paste(median(filter(stroke_ilr,af==1)$time.after.stroke),"(",quantile(filter(stroke_ilr,af==1)$time.after.stroke,0.25),"-",quantile(filter(stroke_ilr,af==1)$time.after.stroke,0.75),")")

paste(median(filter(stroke_ilr,af==1)$time.after.ilr),"(",quantile(filter(stroke_ilr,af==1)$time.after.ilr,0.25),"-",quantile(filter(stroke_ilr,af==1)$time.after.ilr,0.75),")")

paste(median(filter(stroke_ilr,af==1)$chad),"(",quantile(filter(stroke_ilr,af==1)$chad,0.25),"-",quantile(filter(stroke_ilr,af==1)$chad,0.75),")")

paste(median(filter(stroke_ilr,af==1)$bmi),"(",quantile(filter(stroke_ilr,af==1)$bmi,0.25),"-",quantile(filter(stroke_ilr,af==1)$bmi,0.75),")")

paste(median(filter(stroke_ilr,af==1)$duration.holter),"(",quantile(filter(stroke_ilr,af==1)$duration.holter,0.25),"-",quantile(filter(stroke_ilr,af==1)$duration.holter,0.75),")")

paste(median(filter(stroke_ilr,af==1)$mrs.discharge),"(",quantile(filter(stroke_ilr,af==1)$mrs.discharge,0.25),"-",quantile(filter(stroke_ilr,af==1)$mrs.discharge,0.75),")")

paste(median(filter(stroke_ilr,af==1)$mrs.90),"(",quantile(filter(stroke_ilr,af==1)$mrs.90,0.25),"-",quantile(filter(stroke_ilr,af==1)$mrs.90,0.75),")")

paste(median(filter(stroke_ilr,af==1)$mrs.180),"(",quantile(filter(stroke_ilr,af==1)$mrs.180,0.25),"-",quantile(filter(stroke_ilr,af==1)$mrs.180,0.75),")")

paste(median(filter(stroke_ilr,af==0)$age),"(",quantile(filter(stroke_ilr,af==0)$age,0.25),"-",quantile(filter(stroke_ilr,af==1)$age,0.75),")")

paste(mean(filter(stroke_ilr,af==0)$age), "(", sd(filter(stroke_ilr,af==0)$age),")")

paste(median(filter(stroke_ilr,af==0)$admission.nihss),"(",quantile(filter(stroke_ilr,af==0)$admission.nihss,0.25),"-",quantile(filter(stroke_ilr,af==0)$admission.nihss,0.75),")")

paste(median(filter(stroke_ilr,af==0)$days.stroke.ilr),"(",quantile(filter(stroke_ilr,af==0)$days.stroke.ilr,0.25),"-",quantile(filter(stroke_ilr,af==0)$days.stroke.ilr,0.75),")")

paste(median(filter(stroke_ilr,af==0)$days.stroke.af),"(",quantile(filter(stroke_ilr,af==0)$days.stroke.af,0.25),"-",quantile(filter(stroke_ilr,af==0)$days.stroke.af,0.75),")")

paste(median(filter(stroke_ilr,af==0)$days.total),"(",quantile(filter(stroke_ilr,af==0)$days.total,0.25),"-",quantile(filter(stroke_ilr,af==0)$days.total,0.75),")")

paste(median(filter(stroke_ilr,af==0)$ilr.duration),"(",quantile(filter(stroke_ilr,af==0)$ilr.duration,0.25),"-",quantile(filter(stroke_ilr,af==0)$ilr.duration,0.75),")")

paste(median(filter(stroke_ilr,af==0)$time.after.stroke),"(",quantile(filter(stroke_ilr,af==0)$time.after.stroke,0.25),"-",quantile(filter(stroke_ilr,af==0)$time.after.stroke,0.75),")")

paste(median(filter(stroke_ilr,af==0)$time.after.ilr),"(",quantile(filter(stroke_ilr,af==0)$time.after.ilr,0.25),"-",quantile(filter(stroke_ilr,af==0)$time.after.ilr,0.75),")")

paste(median(filter(stroke_ilr,af==0)$chad,na.rm=T),"(",quantile(filter(stroke_ilr,af==0)$chad,0.25,na.rm=T),"-",quantile(filter(stroke_ilr,af==0)$chad,0.75,na.rm=T),")")

paste(median(filter(stroke_ilr,af==0)$bmi,na.rm=T),"(",quantile(filter(stroke_ilr,af==0)$bmi,0.25,na.rm=T),"-",quantile(filter(stroke_ilr,af==0)$bmi,0.75,na.rm=T),")")

paste(median(filter(stroke_ilr,af==0)$duration.holter,na.rm=T),"(",quantile(filter(stroke_ilr,af==0)$duration.holter,0.25, na.rm=T),"-",quantile(filter(stroke_ilr,af==0)$duration.holter,0.75, na.rm=T),")")

paste(median(filter(stroke_ilr,af==0)$mrs.discharge),"(",quantile(filter(stroke_ilr,af==0)$mrs.discharge,0.25),"-",quantile(filter(stroke_ilr,af==0)$mrs.discharge,0.75),")")

paste(median(filter(stroke_ilr,af==0)$mrs.90),"(",quantile(filter(stroke_ilr,af==0)$mrs.90,0.25),"-",quantile(filter(stroke_ilr,af==0)$mrs.90,0.75),")")

paste(median(filter(stroke_ilr,af==0)$mrs.180,na.rm=T),"(",quantile(filter(stroke_ilr,af==0)$mrs.180,0.25,na.rm=T),"-",quantile(filter(stroke_ilr,af==0)$mrs.180,0.75,na.rm=T),")")

**#Comparisons AF yes vs. AF no: categorical variables**

chisq.test(stroke_ilr$af,stroke_ilr$center)

chisq.test(stroke_ilr$af,stroke_ilr$sex)

chisq.test(stroke_ilr$af,stroke_ilr$ethnic)

chisq.test(stroke_ilr$af,stroke_ilr$mRS.prestroke)

chisq.test(stroke_ilr$af,stroke_ilr$hypertension)

chisq.test(stroke_ilr$af,stroke_ilr$strokevsTIA)

chisq.test(stroke_ilr$af,stroke_ilr$cholest)

chisq.test(stroke_ilr$af,stroke_ilr$diabetes)

chisq.test(stroke_ilr$af,stroke_ilr$icd)

chisq.test(stroke_ilr$af,stroke_ilr$chf)

chisq.test(stroke_ilr$af,stroke_ilr$prev.stroke.tia)

chisq.test(stroke_ilr$af,stroke_ilr$prev.antiplatelet)

chisq.test(stroke_ilr$af,stroke_ilr$smoking)

chisq.test(stroke_ilr$af,stroke_ilr$cancer)

chisq.test(stroke_ilr$af,stroke_ilr$oac)

chisq.test(stroke_ilr$af,stroke_ilr$imaging.type)

chisq.test(stroke_ilr$af,stroke_ilr$imaging.vessel)

chisq.test(stroke_ilr$af,stroke_ilr$infarct)

chisq.test(stroke_ilr$af,stroke_ilr$infarct.number)

chisq.test(stroke_ilr$af,stroke_ilr$location)

chisq.test(stroke_ilr$af,stroke_ilr$ant.post)

chisq.test(stroke_ilr$af,stroke_ilr$subtentorial)

chisq.test(stroke_ilr$af,stroke_ilr$left.atrium.abnormal)

chisq.test(stroke_ilr$af,stroke_ilr$mitral.abnormal)

chisq.test(stroke_ilr$af,stroke_ilr$aortic.abnormal)

chisq.test(stroke_ilr$af,stroke_ilr$svt)

chisq.test(stroke_ilr$af,stroke_ilr$ectopics)

chisq.test(stroke_ilr$af,stroke_ilr$mrs.discharge.bad)

chisq.test(stroke_ilr$af,stroke_ilr$mrs.90.bad)

chisq.test(stroke_ilr$af,stroke_ilr$mrs.180.bad)

chisq.test(stroke_ilr$af,stroke_ilr$stroke.recurrent.90)

chisq.test(stroke_ilr$af,stroke_ilr$stroke.recurrent.180)

chisq.test(stroke_ilr$af,stroke_ilr$cv.90)

chisq.test(stroke_ilr$af,stroke_ilr$cv.180)

chisq.test(stroke_ilr$af,stroke_ilr$composite.90)

chisq.test(stroke_ilr$af,stroke_ilr$composite.180)

**#Comparisons AF yes vs. AF no: continuous variables**

wilcox.test(age~af,stroke_ilr)

wilcox.test(admission.nihss~af,stroke_ilr)

wilcox.test(days.stroke.ilr~af,stroke_ilr)

wilcox.test(days.total~af,stroke_ilr)

wilcox.test(time.after.stroke~af,stroke_ilr)

wilcox.test(time.after.ilr~af,stroke_ilr)

wilcox.test(chad~af,stroke_ilr)

wilcox.test(bmi~af,stroke_ilr)

wilcox.test(duration.holter~af,stroke_ilr)

wilcox.test(mrs.discharge~af,stroke_ilr)

wilcox.test(mrs.90~af,stroke_ilr)

wilcox.test(mrs.180~af,stroke_ilr)

wilcox.test(mRS.prestroke~af,stroke_ilr)

**#Cox univariate regression for AF prediction**

**Packages:** library(“survminer”), library (“survival”)

**#Cox multivariate regression for AF prediction**

**Packages:** library(“survminer”), library (“survival”)

**#Cumulative incidence of AF in ESUS patients with vs. without subtentorial involvement**

**Packages:** library("ggplot2"), library("ggcuminc")

cuminc(Surv(time.after.stroke,af)~subtentorial, data=stroke_ilr) %>% ggcuminc() +labs(x="Days", y="Cumulative AF incidence after stroke") + add_risktable() +add_pvalue() +scale_fill_discrete(labels=c("Supratentorial","Subtentorial")) + add_confidence_interval("ribbon","ribbon") +scale_color_discrete(labels=c("Supratentorial","Subtentorial")) +coord_cartesian(xlim=c(0,950))
